# Improved intestinal absorption of paclitaxel by mixed micelles self-assembled from vitamin E succinate-based amphiphilic polymers and their transcellular transport mechanism and intracellular trafficking routes

**DOI:** 10.1080/10717544.2017.1419513

**Published:** 2018-01-09

**Authors:** Xiaoyou Qu, Yang Zou, Chuyu He, Yuanhang Zhou, Yao Jin, Yunqiang Deng, Ziqi Wang, Xinru Li, Yanxia Zhou, Yan Liu

**Affiliations:** ^a^ Beijing Key Laboratory of Molecular Pharmaceutics and New Drug Delivery Systems, School of Pharmaceutical Sciences, Peking University Beijing China

**Keywords:** Paclitaxel, mixed polymeric micelles, intestinal absorption, intracellular trafficking, transcytosis mechanism

## Abstract

To ensure that antitumor drugs can be effectively transported across intestinal barrier and then quickly released in tumor cells, mixed polymeric micelles (Mix-PMs) were designed and fabricated by combining poly(2-ethyl-2-oxazoline)-vitamin E succinate (PEOz-VES) with TPGS1000 for enhancing intestinal absorption of paclitaxel. PEOz-VES exhibited an extremely low critical micelle concentration and negligible cytotoxicity. The Mix-PMs were characterized to have about 20 nm in diameter, uniform spherical morphology, high drug-loading content and sustained drug release profile with a retained pH-sensitivity. The results of the transport through Caco-2 cell monolayers and intestinal absorption revealed that Mix-PMs displayed higher transcellular transport efficiency compared with PEOz-VES micelles and Taxol^®^. The possible mechanism of transcellular transport for Mix-PMs was elucidated to be mainly through clathrin- and caveolae/lipid rafts-mediated transcytosis. Confocal laser scanning micrographs revealed that late endosomes, lysosomes, endoplasmic reticulum, Golgi apparatus, and mitochondria were all involved in intracellular trafficking of Mix-PMs. The proteins involved in transcytosis of Mix-PMs and finally excreted were unraveled for the first time by the analysis of proteins in the basolateral media according to the proteomics method. Consequently, the fabricated mixed polymeric micelles may have great potential in enhancing intestinal absorption and accelerating drug release in tumor cells.

## Introduction

Oral administration is the most readily accepted and widely used route of drug delivery because of its convenience and low cost, especially for chronic therapies (Zhang et al., 2013). Also, it allows controlled and flexible dosing schedule. However, oral administration of BCS Class IV drugs still faces great challenges due to their poor solubility in the gastrointestinal (GI) tract and low permeability across the intestinal epithelium barrier. For example, poor water solubility and permeability through intestinal epithelium, P-glycoprotein (P-gp) mediated efflux, and extensive metabolism of paclitaxel (PTX), a typical BCS Class IV drug, have seriously constrained its application as oral chemotherapy (He et al., 2013; Simoes et al., 2015). Nowadays, the development of nanotechnology in drug delivery areas has extremely altered the traditional comprehension on orally administered formulations and made it possible to overcome these barriers to some extent (He et al., 2013). Polymeric micelles have attracted much attention and presented great potential in effective delivery of these drugs through gastrointestinal tract (Zhang et al., 2010a,b; Lian et al., 2013; Simoes et al., 2015).

As known, polymeric micelles with core-shell structure are self-assembled from amphiphilic polymers. The inner core consisted of hydrophobic chains of the polymers can encapsulate poorly water-soluble drugs while the outer corona formed by the hydrophilic chains of the polymers protects drugs from degradation in biological media such as GI fluid (Dahmani et al., 2012). Further, drug-loaded polymeric micelles with nanoscaled size (∼100 nm) are in favor of transport of drugs across intestinal epithelium (Zhang et al., 2010a,b) and promotion of endocytosis and transcytosis (Plapied et al., 2011; Simoes et al., 2015). Generally, well-designed polymeric micelles for oral delivery of anticancer drugs should have such characteristics as tumor targeting and rapid drug release inside tumor cells besides favorable stability in GI tract as well as enhanced permeability through intestinal epithelium. However, previous documents on the polymeric micelles for oral delivery of anticancer drugs mainly focused on drug solubilization and transcellular transport (Kim et al., 2008; Wang et al., 2015). Consequently, there is an urgent need to construct desired micelles to make sure that the encapsulated antitumor drugs are orally delivered to tumor sites and rapidly released inside the tumor cells to take effect.

Our previous work demonstrated that polymeric micelles based on diblock copolymers with poly(2-ethyl-2-oxazoline) (PEOz) hydrophilic segment exhibited pH-triggered drug release behavior, which is beneficial for enhancement of anticancer efficacy by passive targeting delivery of anticancer drugs to tumor sites and quick release of the loaded drugs in tumor cells (Gao et al., 2015a,b,c; Wang et al., 2017). Furthermore, vitamin E succinate (VES) has been proved to be an effective anticancer agent with high selectivity for malignant cells without toxicity to normal cells (Duhem et al., 2014). In addition, better chemical compatibility of PTX with VES is beneficial for entrapment of PTX due to the fact that aromatic groups are present in their molecular structures (Lavasanifar et al., 2002; Liu et al., 2004). Thus, it is considered to be a desired component of the inner core of PTX-loaded micelles. Therefore, a new amphiphilic polymer PEOz-VES consisting of PEOz as a hydrophilic segment and VES as a hydrophobic segment was designed as micelle-forming component. Furthermore, d-α-tocopherol polyethylene glycol 1000 succinate (TPGS1000), another amphiphilic material with hydrophobic segment of VES, approved by US FDA as a safe pharmaceutical adjuvant, has intrinsic anticancer activity (Duhem et al., 2014). Previous documents reported that TPGS1000 can be used as a P-gp efflux inhibitor for overcoming multidrug resistance to enhance antitumor efficacy and improving oral bioavailability of antitumor drugs (Dintaman & Silverman, 1999; Zhang et al., 2012). Unfortunately, the critical micelle concentration (CMC) of TPGS1000 is relatively high (200 mg/L) (Wu & Hopkins, 1999), implying that TPGS1000 micelles are easily breaking apart upon dilution in biological media. Hence, TPGS1000 is usually combined with other amphiphilic polymers to construct mixed micelles for improving micelle stability and drug encapsulation (Zhao & Yung, 2009; Gao et al., 2015c; Zhao et al., 2015).

Furthermore, the studies on the transcellular transport pathway and intracellular trafficking are very important to design desirable nanosized drug carriers. But so far, previous researches mainly focused on intracellular trafficking of polymeric micelles following internalization by tumor cells, little is known about transcellular trafficking route of polymeric micelles across Caco-2 cell monolayers.

To address these issues in the present work, the merits of PEOz-VES for its extremely low CMC and pH-sensitivity and TPGS1000 for its P-gp inhibition activity were integrated to fabricate newly mixed polymeric micelles for enhancing transmembrane transport and accelerating release inside the tumor cells of PTX in turn. Therefore, the physicochemical properties of PTX-loaded mixed polymeric micelles including particle size, drug loading, morphology and *in vitro* release were characterized. Their cytotoxicity against Caco-2 cells was evaluated. In addition, the potential transcellular transport pathways and intracellular trafficking routes of the mixed polymeric micelles were disclosed. The constructed mixed micelles were hoped to be efficient intestinal delivery carriers of antitumor drugs.

## Materials and methods

### Materials

2-Ethyl-2-oxazoline (EOz) and vitamin E succinate (VES) were purchased from TCI Development Co., Ltd. (Tokyo, Japan). Paclitaxel (PTX) was obtained from Guilin Huiang Biopharmaceutical Co. Ltd. (Guilin, China). 1-[3-(Dimethylamino)propyl]-3-ethylaarbodiimide hydrochloride (EDC·HCl) and N-Hydroxysuccinimide (NHS) were obtained from J&K chemical Co., Ltd. (Beijing, China). 3-(4,5-dimethyl-thiazol-2-yl)-2,5-diphenyl tetrazolium bromide (MTT) and sodium deoxycholate (DOC) were gained from Amresco (USA). TPGS1000, chlorpromazine, methylated-β-Cyclodextrin (M*β*CD), amiloride, genistein, brefeldin A, and trichloroacetic acid (TCA) were all purchased from Sigma (St. Louis, MO). Lyso-Tracker Red DND-99, Mito-Tracker Deep Red, ER-Tracker Red, Golgi-Tracker BODIPY-TR ceramide, and CellLight Late Endosome-RFP were all obtained from Invitrogen (Carlsbad, CA). Dulbecco’s modified Eagle’s medium (DMEM), Hank’s balanced salt solution (HBSS), penicillin–streptomycin, non-essential amino acids, and trypsin-EDTA were all obtained from MAC Gene Technology (Beijing, China). Fetal bovine serum (FBS) was supplied by GIBCO (Burlington, Ontario).

### Animals

Normal male SD rats weighing 200–240 g were obtained from the Animal Center of the Peking University Health Science Center. All care and handling of animals were performed with the approval of Institutional Authority for Laboratory Animal Care of the Peking University.

### Synthesis and characterization of PEOz-VES polymer

**Figure 1.**
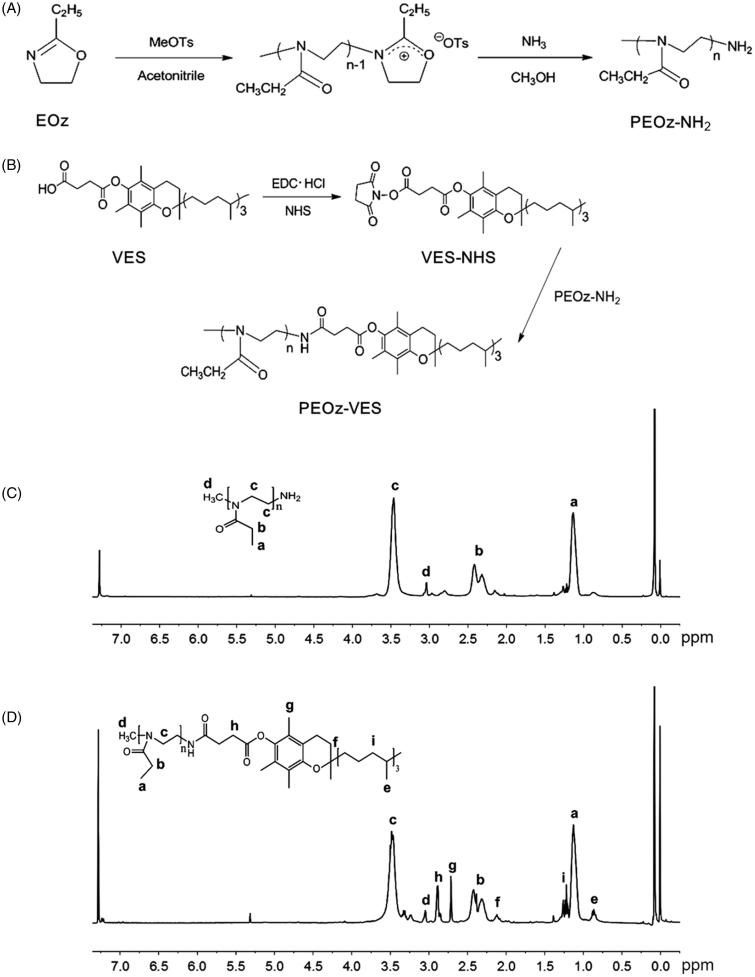
Synthetic routes of PEOz-NH_2_ (A) and PEOz-VES (B) copolymers. ^1^H NMR spectrum of PEOz-NH_2_ (C) and PEOz-VES in CDCl_3_ (D).

PEOz-VES polymer was synthesized by two steps. First, amino terminated poly(2-ethyl-2-oxazoline) (PEOz-NH_2_) was synthesized through cationic ring-opening polymerization of EOz (20 mL) initiated by methyl *p*-toluenesulfonate (MeOTs) (10:1 molar ratio of EOz to MeOTs) in acetonitrile (40 mL) at 100 °C for 24 h under nitrogen atmosphere and then terminated by methanol saturated with ammonia (10:1 molar ratio of NH_3_ to MeOTs) at 170 °C for 24 h in a high-pressure reactor (Figure 1(A)) (Wang et al., 2009). After cooling down to ambient temperature, the reaction was quenched by adding K_2_CO_3_ (10:1 molar ratio of K_2_CO_3_ to MeOTs) and stirred at room temperature for 24 h to remove extra ammonia. The salt was removed by filtration of the resulting mixture through Sabouraud funnel, and the solvent was then removed by rotary evaporation. The obtained crude product was purified by dissolving in dichloromethane and then recrystallization with cold ethyl ether for several times. A white powder solid of PEOz-NH_2_ was obtained by vacuum drying.

Next, to the solution of EDC·HCl (2.88 g), NHS (2.16 g), and VES (6.64 g) in dichloromethane (60 mL) being stirred for 2 h in ice bath, the solution of PEOz-NH_2_ (10.00 g) in dichloromethane (40 mL) was added, then stirred at ambient temperature for 48 h (Figure 1(B)). Finally, the pure product of PEOz-VES was obtained by recrystallization with cold ethyl ether for several times.

The composition of the obtained polymers was analyzed by ^1^H NMR spectra (Bruker MSL2300 spectrometer, Germany) in CDCl_3_. The molecular weight and polydispersity of the products were analyzed by gel permeation chromatography (GPC, Spectra System P100).

### Determination of *p*K_a_ of PEOz-NH_2_ and PEOz-VES

The p*K*_a_ of PEOz-NH_2_ and PEOz-VES was measured by use of acid-base titration as our previous report (Zhao et al., 2015; Wang et al., 2017).

### Measurement of critical micelle concentration

The critical micelle concentration (CMC) of PEOz-VES and a mixture of PEOz-VES with TPGS1000 in a mass ratio of 1:1 was measured using a fluorescence technique as reported earlier (Li et al., 2009; Gao et al., 2015a,b; Zhao et al., 2015).

### Preparation of polymeric micelles

PTX-loaded PEOz-VES polymeric micelles (denoted as PTX/PV-PMs) were prepared using film hydration method with a little modification (Gao et al., 2015a,b). Briefly, the solution of PEOz-VES (30 mg) and PTX (1 mg) in methanol (10 mL) was rotationally evaporated under vacuum at 30 °C to form a thin stripped film. Then 10 mL of deionized water was added to hydrate the resulting film at 60 °C followed by vortexing for 5 min. A clear and homogeneous micelle solution was finally obtained by filtration through a 0.22 μm filter to remove non-encapsulated PTX.

PTX-loaded PEOz-VES/TPGS1000 mixed polymeric micelles (denoted as PTX/Mix-PMs) were prepared as mentioned earlier except that PEOz-VES was replaced by a mixture of PEOz-VES and TPGS1000 in a mass ratio of 1:1.

Coumarin-6 (C6)-, DiI-, DiO-, and DiO/DiI-loaded mixed polymeric micelles were also prepared as mentioned earlier. The C6/polymer ratio for C6-loaded mixed micelles (denoted as C6/Mix-PMs) was 1:1000 (w/w). The DiO/polymer ratio for DiO-loaded mixed micelles (denoted as DiO/Mix-PMs) was 1:500 (w/w), DiI/polymer ratio for DiI-loaded mixed micelles (denoted as DiI/Mix-PMs) was 1:1000 (w/w), and DiO/DiI/polymer ratio for (DiO/DiI)-loaded micelles (denoted as FRET micelles) were 2:1:1000 (w/w).

### Physicochemical characterization of polymeric micelles

Dynamic light scattering (DLS, Malvern Zetasizer Nano ZS, UK) was used to determine the size and its distribution, and Zeta potential of polymeric micelles diluted with deionized water (Gao et al., 2015a). The morphology of micelles was observed via a transmission electron microscope (TEM, JEM-1230, JEOL, Japan) (Gao et al., 2015a,b; Zhao et al., 2015; Wang et al., 2017). The encapsulation efficiency (EE) and loading content (LC) of the micelles were determined as previously reported (Li et al., 2010; Gao et al., 2015a,b; Wang et al., 2017).

### *In vitro* release of PTX from polymeric micelles

The *in vitro* release of PTX from the micelles was evaluated using a dialysis diffusion technique as previously described except that PBS (pH 5.0, 6.5, and 7.4) and the simulated intestinal fluid (SIF) with 0.2% Tween 80 were selected as release medium, respectively (Gao et al., 2015a,b; Zhao et al., 2015; Wang et al., 2017). At pre-determined time point, 1 mL of the release medium was withdrawn and immediately replaced with 1 mL of fresh medium. The concentration of PTX in release medium was determined using the HPLC method as mentioned earlier.

### Intestinal absorption of PTX-loaded polymeric micelles

Intestinal absorption of PTX-loaded polymeric micelles was assayed by *in situ* single-pass intestinal perfusion method (Song et al., 2006; Li et al., 2010; Zhang et al., 2010a,b). In brief, prior to the experiments, rats were fasted for 12 h but allowed free access to water, and then anesthetized through intraperitoneal injection of 20% (w/v) urethane at a dose of 1 g/kg. Afterward, the abdominal cavity was opened, and the small intestine segment was exposed and gently rinsed with warm saline solution to clear the content by use of constant flow pump. The surgical area was covered with pledget soaked with 37 °C saline solution to avoid dehydration, and the normal body temperature of the rats was kept by use of a heating lamp during the whole period of the experiment. Then the intestinal segment was flushed for 10 min with Krebs–Ringer’s buffer (KRB) at a flow rate of 0.2 mL/min. Drug perfusion solution (containing 30 μg/mL PTX for each tested sample in KRB with 20 μg/mL phenol red, a non-absorbable marker to correct the appreciable effect of the secretion/absorption of water on PTX content during the whole period of the experiment) was then infused at a flow rate of 0.2 mL/min and the time was set 0 just as the beginning of the perfusion. When steady-state was reached after 30 min, the perfused samples were collected every 15 min up to 120 min, frozen immediately and stored at −20 °C for analysis. In addition, at the end of the experiment, the length of intestinal segment was measured after the rats were euthanized.

For the sample analysis, 0.2 mL perfusate was mixed with 0.8 mL methanol and then centrifuged at 10,000 rpm for 10 min. The supernatant was then analyzed by HPLC method to determine the content of PTX in perfusate. In addition, the mixed solution of 0.1 mL perfusate with 0.9 mL NaOH (0.1 M) was used to measure the content of phenol red by UV spectrophotometer (Agilent 8453, Agilent Technologies, UK) at 558 nm. The effective permeability (*P*_eff_) was calculated as follows (Cook & Shenoy, 2003; Song et al., 2006):

$$P_{\text{eff}}=-\frac{Q}{2\pi rL}\ln(\frac{C_{\text{out}}}{C_{\text{in}}}\times \frac{{\text{PR}}_{\text{in}}}{{\text{PR}}_{\text{out}}})$$

where *Q* represents the perfusion flow rate (0.2 mL/min), *r* represents the radius of the intestine (0.18 cm), *L* represents the length of the perfused intestinal segment (cm), *C*_in_ and *C*_out_ are PTX concentrations in the inlet and outlet perfusate, respectively, PR_in_ and PR_out_ are the inlet and outlet concentrations of phenol red, respectively.

### *In vitro* cytotoxicity assessment

The Caco-2 cells were seeded in 96-well plates at a density of 1 × 10^4^ cells/well and incubated with fresh serum-free medium containing various blank PV-PMs, TPGS1000-PMs and Mix-PMs with serial concentration of polymers. After incubation for 6 h, the cell viability was evaluated using MTT assay (Qiu et al., 2014).

Further, the cytotoxicity of PTX/PV-PMs and PTX/Mix-PMs against Caco-2 cells was also evaluated as described above except that the PTX-loaded micelles were incubated with Caco-2 cells for 3 h.

### Micelle integrity analysis after being internalized by Caco-2 cells

The integrity of the micelle structure was assessed through the release of hydrophobic fluorescent probes loaded in micelles using FRET method after they were internalized by Caco-2 cells (Yu et al., 2013; Wang et al., 2017). Briefly, Caco-2 cells were seeded on a 35-mm glass bottom culture dish at a density of 2 × 10^5^ cells per dish and cultured with the medium containing DiO/Mix-PMs, DiI/Mix-PMs and FRET micelles (the final concentration of DiO and DiI was 6 and 3 μg/mL, respectively). After incubation for 2, 4, and 6 h at 37 °C, respectively, the cells were washed thrice with cooled PBS, fixed with 4% paraformaldehyde for 15 min at 37 °C, rinsed thrice with PBS, and sealed with glycerin jelly. Then FRET images were observed with a confocal laser scanning microscope (CLSM, TCS SP5, Leica, Germany) after the crosstalk was corrected with DiO/Mix-PMs- and DiI/Mix-PMs-treated groups, respectively. The excitation/emission wavelength for DiO was 488 nm/500–530 nm, and 549 nm/555–655 nm for DiI. The excitation wavelength (488 nm) of DiO as the donor and the emission wavelength (568 nm) of DiI as the acceptor were used, respectively, for imaging the FRET lines (Gao et al., 2015a).

### Transport studies of drug-loaded polymeric micelles across Caco-2 cell monolayers

Caco-2 cell monolayers were obtained by seeding Caco-2 cells at a density of 2 × 10^5^ cells/well on Transwell^®^ polycarbonate membrane inserts and culturing for about 21 days after seeding (Zhang et al., 2010a,b; Li et al., 2014). The culture medium was refreshed every other day as the apical (AP) and basolateral (BL) compartments received 0.5 and 1.5 mL of culture medium, respectively. The transepithelial electrical resistance (TEER) was monitored with an electrical resistance meter (Millicell ERS-2, Millipore) to detect the integrity of cell monolayers. Cell monolayers with TEER values higher than 300 Ω/cm^2^ were considered to be integrated and intact, and could be used for experiments (Wang et al., 2015).

For transcellular transport studies, the culture medium in both sides was removed and the monolayer was washed thrice with pre-warmed HBSS and allowed to equilibrate with HBSS at 37 °C for 30 min. Then 0.5 mL of tested solutions of PTX/PV-PMs, PTX/Mix-PMs, and Taxol^®^ in HBSS with equivalent concentrations of 20 μg/mL PTX was added to AP side, and 1.5 mL of fresh HBSS was added to BL side. At pre-determined time points of 0.5, 1, 1.5, 2, and 3 h after administration, all of HBSS was withdrawn from BL side, and 1.5 mL of pre-warmed fresh HBSS was then supplemented. The withdrawn sample was lyophilized, followed by addition of 200 μL methanol, vortexing for 3 min and centrifugation for 5 min. The concentration of PTX in supernatant was assayed by HPLC method as mentioned above. The apparent permeability coefficient (*P*_app_) for PTX in various formulations was calculated as follows:

$$P_{\text{app}}=\frac{\text{d}Q}{\text{d}t}\times \frac{1}{AC_{0}}$$

where d*Q*/d*t* represents the flux of PTX from AP side to BL side, *A* represents the surface area of the membranes, and *C*_0_ represents the initial concentration of PTX in the AP compartment (Wang et al., 2015). Furthermore, the integrity of cell monolayers was monitored with TEER value during the whole period of experiments.

### Transcytosis pathway exploration of polymeric micelles

To investigate whether the transcellular transport of Mix-PMs is energy-dependent, Caco-2 cell monolayers were pre-incubated at 4 °C for 30 min and then treated with PTX/Mix-PMs with a final PTX concentration of 20 μg/mL for another 3 h at 4 °C. The content of transported PTX was determined and the *P*_app_ values were calculated accordingly as described earlier.

To explore the possible transcytosis pathway of mixed polymeric micelles, the Caco-2 cell monolayers were pre-incubated at 37 °C with 0.5 mL of HBSS containing various endocytosis inhibitor such as chlorpromazine hydrochloride (10 μg/mL), methyl-*β*-cyclodextrin (M*β*CD) (10 mM), genistein (100 μM) and amiloride (0.1 mM), and Golgi related inhibitor brefeldin A (BFA, 25 μg/mL) for 30 min, respectively. After the pre-incubation, the solution at the AP side was replaced with PTX/Mix-PMs in HBSS with the corresponding inhibitors and incubated for another 3 h at 37 °C (Mo et al., 2011; He et al., 2013; Abramov et al., 2015). Then, the amount of transported PTX was determined and *P*_app_ values were calculated.

### Intracellular trafficking of polymeric micelles tracked by confocal microscope

The location of mixed polymeric micelles inside the Caco-2 cells was analyzed using organelle trackers (Supplementary Table S1) and C6/Mix-PMs in living cell condition. Caco-2 cells were first incubated with different organelle trackers for a certain time at 37 °C according to the instructions, including Endo-Tracker probe, Lyso-Tracker probe, ER-Tracker probe, Golgi-Tracker and Mito-Tracker probe, and then the medium was replaced with C6/Mix-PMs solution in HBSS at 37 °C. After 1 h of incubation, the medium was replaced with fresh HBSS. Then the cells were imaged using CLSM. Pearson’s correlation coefficient and colocalization rate which are related to colocalization were obtained through analysis of the obtained images using quantitative software (LEICA TCS SP8).

### Analysis of proteins involved in transcytosis of mix-PMs and finally excreted

The Caco-2 cell monolayer was washed three times with HBSS and pre-equilibrated for 30 min with HBSS. Then 500 μL of 4 mg/mL Mix-PMs solution in HBSS was added to the AP side, and 1.0 mL of fresh HBSS was added to the BL side. After incubation for 3 h, all of basolateral media were collected, concentrated and analyzed. For the control group, the experimental procedures were the same as the tested group except that 500 μL of 4 mg/mL Mix-PMs solution in HBSS was replaced with 500 μL of fresh HBSS for the AP side.

The deoxycholate–trichloroacetic acid method was used to concentrate and precipitate the secreted proteins (Bensadoun & Weinstein, 1976; Koontz, 2014). Briefly, 2.5 mL of 0.15% sodium deoxycholate was added to 25 mL of the sample solution in a centrifugation tube, and then the mixture was vortexed for 10 min at room temperature, followed by addition of 1.25 mL of 100% TCA and vortexing. The precipitate was recovered by centrifuging at 10,000 *g* for 20 min at 4 °C. The precipitated proteins were dissolved by vortexing in a minimal volume of 0.1 M NaOH to neutralize the excess acid. After being quantitated by BCA protein assay kit, the protein solution was mixed with SDS loading buffer and heated in water bath at 100 °C for 5 min, and then purified and concentrated by SDS-PAGE. After in-gel digestion, the sample was analyzed by mass spectrometry (LTQ velos pro, Thermo) and Thermo Proteome Discoverer software. The proteins involved in transcytosis of Mix-PMs and finally excreted to basolateral media were obtained by comparison of the results of Mix-PMs-treated group with the control group, and homed by using the UniProtKB database. In general, the result is considered to be highly reliable as the score is greater than 20.

### Statistical analysis

The statistical significance of differences among more than two groups was analyzed using one-way ANOVA. A statistically significant difference was set at *p* ≤ .05.

## Results

### Synthesis and characterization of PEOz-VES

PEOz-NH_2_ was synthesized by the cationic ring-opening polymerization of EOz by MeOTs and the living end was then terminated by NH_3_ (Figure 1(A)). The composition of PEOz-NH_2_ was confirmed by ^1^H NMR spectrum in CDCl_3_ (Figure 1(C)). The sharp peaks at 2.22 and 1.14 ppm belonged to methylene and methyl protons in the side chains, respectively. The peak at 3.46 ppm was attributed to the methylene protons in the backbone. The little peak at 3.04 ppm was assigned to the methyl protons at the end of the polymer. These were consistent with the previous document (Wang et al., 2009). The number-average molecular weight (*M*_n_) of the synthesized PEOz-NH_2_ measured by GPC was 897 g/mol with 1.30 of PDI.

PEOz-NH_2_ was then reacted with VES to form PEOz-VES (Figure 1(B)). The characteristic chemical shifts corresponding to VES (2.95, 2.56, 1.98, and 0.86 ppm) and PEOz (3.46 and 1.14 ppm) were identified in Figure 1(D) implying the successful synthesis of PEOz-VES. The *M*_n_ of PEOz-VES measured by GPC was 1212 g/mol with 1.50 of PDI.

As known, a CMC of less than 135 mg/L has been considered to be an indicative guide to resist rapid dissociation upon oral administration (Gaucher et al., 2010). Therefore, the CMC is an effective parameter of micelle stability *in vivo*. The CMC of PEOz-VES was determined to be 5.84 mg/L (Supplementary Figure S1A), implying that the micelles formed by PEOz-VES might exhibit better diluting stability in GI tract and systemic circulation. In contrast, the CMC of TPGS1000 is about 200 mg/L, it is therefore combined with other amphiphilic polymers to form mixed micelles for increasing diluting stability and drug encapsulation (Duhem et al., 2014).

The appropriate composition of mixed micelles was optimized to be 1:1 of mass ratio of PEOz-VES with TPGS1000 according to preliminary tests of *in vitro* pH-dependent release. The CMC of this mixed system was determined to be 13.98 mg/L (Supplementary Figure S1B). Hence, the mixed micelles prepared in the present study might be suitable for oral delivery of drugs.

The p*K*_a_ of PEOz-NH_2_ and PEOz-VES were determined to be 7.21 and 6.01, respectively (Supplementary Figure S2). When pH value in the external environment is lower than p*K*_a_ of PEOz-VES, the amide groups of PEOz will be ionized (Zhao et al., 2015), making PEOz-VES be a pH-responsive polymer.

### Preparation and physicochemical characterizations of polymeric micelles

**Figure 2.**
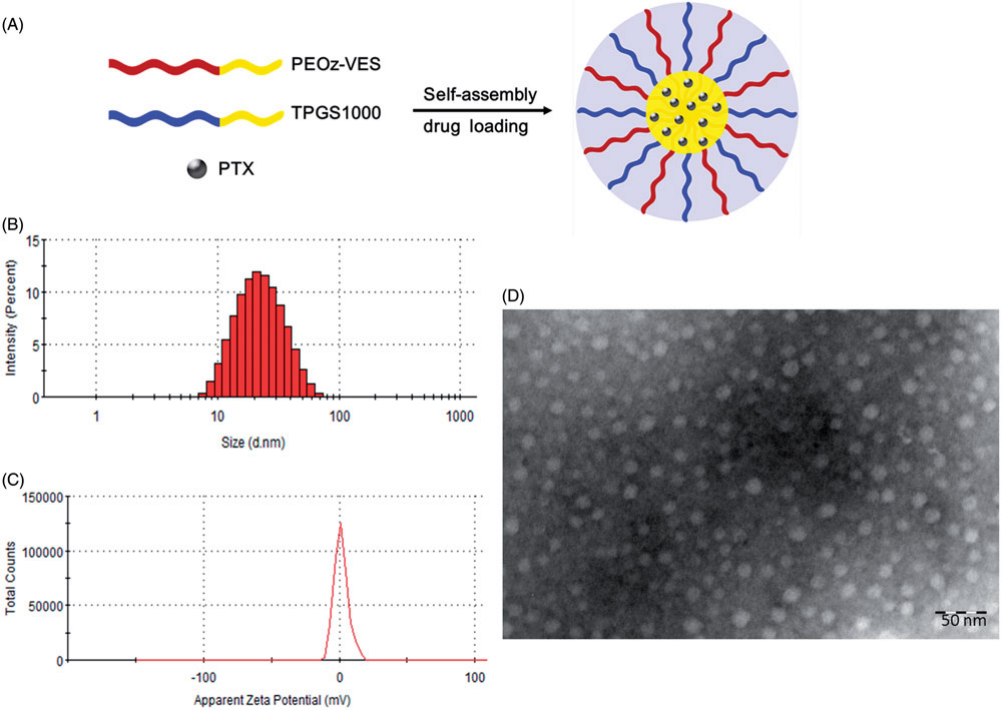
(A) Schematic illustration of self-assembly for PTX/Mix-PMs in aqueous medium. Size (B), Zeta potential (C), and transmission electron microscope images (D) of PTX/Mix-PMs.

A schematic diagram depicting the self-assembly and drug loading of mixed micelles was shown in Figure 2(A). As known, the physicochemical characteristics of polymeric micelles play an important role in determining their *in vivo* fate. The prepared polymeric micelles were therefore characterized by their mean size and Zeta potential. PTX/Mix-PMs was about 20 nm in diameter (Figure 2(B) and Supplementary Table S2), suggesting that these micelles were beneficial for transport across intestinal barrier and passive tumor targeting delivery of drugs loaded in micelles in turn through enhanced permeability and retention (EPR) effect, while reducing reticuloendothelial system (RES)-mediated clearance and avoiding renal filtration after crossing intestinal barrier (Duan & Li, 2013). TEM images presented a visual evidence for the formation of PTX/Mix-PMs with a well-defined spherical shape (Figure 2(D)). Notably, addition of TPGS1000 into PV-PMs apparently led to significant reduction in micelle size (*p* < .001) and significant increase in EE and LC (*p* < .01) (Supplementary Table S2) without visible influence on micelle morphology. Additionally, both PTX/PV-PMs and PTX/Mix-PMs exhibited a slightly positive Zeta potential (Figure 2(C) and Supplementary Table S1). These results suggested that Mix-PMs had better physicochemical characteristics compared with PV-PMs.

**Figure 3.**
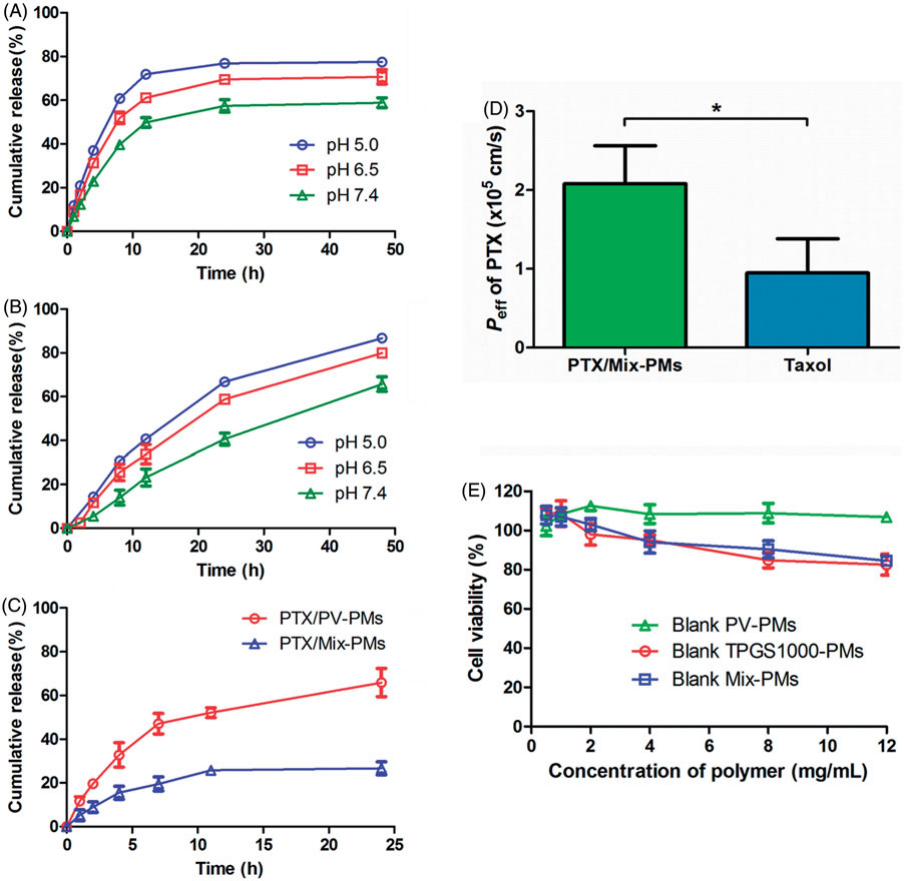
*In vitro* release profiles of PTX from PTX/PV-PMs (A) and PTX/Mix-PMs (B) in PBS with 0.2% Tween 80 and in simulated intestinal fluid (SIF) with 0.2% Tween 80 (C) at 37 °C (*n* = 3). (D) The effective permeability of PTX/Mix-PMs and Taxol^®^ in rat intestine (*n* = 3). *p < .05. (E) Cytotoxicity of blank PV-PMs, TPGS1000-PMs and Mix-PMs to Caco-2 cells after incubation for 6 h (*n* = 6).

The *in vitro* release behavior of PTX from polymeric micelles at 37 °C was first investigated in PBS with pH 7.4 that mimics the blood environment, in PBS with pH 6.5 that stimulates the extracellular microenvironment of tumor and under endo/lysosome mimetic circumstance (PBS, pH 5.0) using a dialysis method. As shown in Figure 3(A,B), the release of PTX from both PV-PMs and Mix-PMs displayed pH-dependent pattern and was gradually accelerated with decrease of pH value. For PTX/PV-PMs (Figure 3(A)), the release of PTX was about 49.9% at pH 7.4, 61.1% at pH 6.5, and 71.9% at pH 5.0 within the first 12 h, respectively, and then the PTX release profiles reached a plateau. These results suggested that PTX/PV-PMs could distinguish endo/lysosomal pH and tumor extracellular pH from physiological pH by accelerating drug release. As for PTX/Mix-PMs (Figure 3(B)), the release of PTX was obviously delayed compared with PTX/PV-PMs. The release of PTX was about 23.3% at pH 7.4, 33.8% at pH 6.5, and 40.7% at pH 5.0 within the first 12 h, respectively, and PTX release was sustained thereafter. Based on these results, it could be concluded that both PV-PMs and Mix-PMs could rapidly release drugs inside the tumor cells following their internalization into tumor cells and PV-PMs exhibited better. But on the other hand, these results also suggested that PV-PMs were not used for gastrointestinal delivery of drugs, whereas Mix-PMs might be appropriate for intestinal administration.

Generally, it is essential for developing oral drug delivery systems for poorly water-soluble drugs to adequately control drug release to avoid drug precipitation upon dilution in GI tract and maximize intestinal absorption. The *in vitro* release of PTX from polymeric micelles in simulated intestinal fluid (SIF) was then therefore investigated and PTX/PV-PMs were used as control. The release profile of PTX/Mix-PMs profoundly differed from that of PTX/PV-PMs (Figure 3(C)). Specifically, the release of PTX from PTX/PV-PMs and PTX/Mix-PMs was about 32.8 and 15.5% within the first 4 h, and about 52.2 and 25.8% within 11 h, respectively. Further, the PTX release profile reached a plateau after 11 h for PTX/Mix-PMs, whereas the release was sustained thereafter for PTX/PV-PMs. These implied that the stability of the micelles in intestine was greatly improved by insertion of TPGS1000 into PV-PMs and was favorable for intestinal administration.

### Intestinal absorption of PTX-loaded mixed micelles

The intestinal absorption of PTX/Mix-PMs was evaluated based on intestine permeability of PTX in rat intestine segments. As shown in Figure 3(D) the effective permeability *P*_eff_ of PTX/Mix-PMs [(2.08 ± 0.39) × 10^−5 ^cm/s] was significantly (2.19-fold) higher than that of commercial formulation Taxol^®^ [(0.95 ± 0.35) × 10^−5 ^cm/s] (*p* < .05), suggesting that the intestinal absorption of PTX was evidently enhanced by Mix-PMs compared with Taxol^®^. In addition, Taxol^®^ with the *P*_eff_ being in the range of 0.3 × 10^−5^–2 × 10^−5 ^cm/s was considered to be moderate absorption, whereas PTX/Mix-PMs with *P*_eff_ > 2 × 10^−5 ^cm/s was regarded as complete absorption (Fagerholm et al., 1996; Zakeri-Milani et al., 2005).

### Transport through Caco-2 cell monolayers

Biocompatibility is a great concern for biomedical materials. In order to address the cytocompatibility of micelle-forming materials with Caco-2 cells, their cytotoxicity toward Caco-2 cells by MTT assay was therefore assessed. As shown in Figure 3(E) a relative cell viability ranging from (102.3 ± 5.0)% to (112.5 ± 2.3)% was observed for blank PV-PMs up to the concentration of 12 mg/mL PEOz-VES, suggesting that PEOz-VES exhibited excellent biocompatibility with cells and was a favorable micelle-forming biomaterial for drug delivery. In contrast, the cell viability for blank TPGS1000-PMs-treated group was higher than 82% at concentrations ranging from 0.5 to 12 mg/mL TPGS1000, indicating that TPGS1000 also had better biocompatibility with cells, but it was a little inferior to PEOz-VES. Similarly, the blank Mix-PMs exhibited almost no toxicity against Caco-2 cells up to a concentration of 12 mg/mL polymer. In conclusion, PEOz-VES exhibited higher safety compared with TPGS1000, and the influence of blank PV-PMs and Mix-PMs on cell viability could be ignored in the subsequent studies.

**Figure 4.**
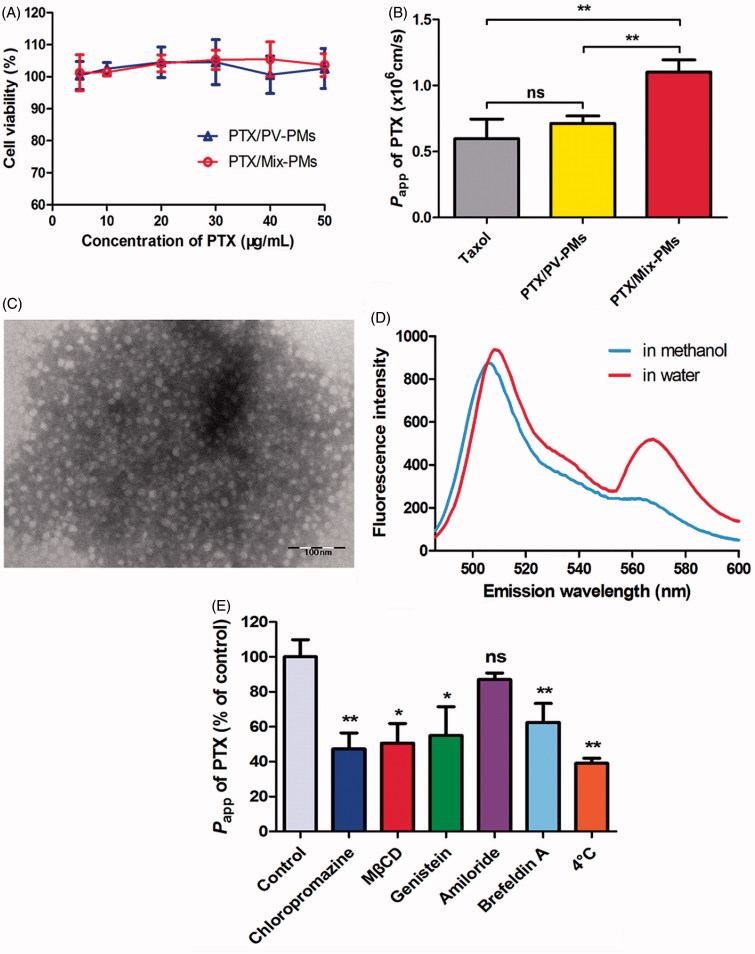
(A) Cytotoxicity of PTX/PV-PMs and PTX/Mix-PMs to Caco-2 cells after incubation for 3 h (*n* = 6). (B) Apparent permeability coefficients of PTX for different formulations (Taxol^®^, PTX/PV-PMs, and PTX/Mix-PMs) at 20 μg/mL PTX across Caco-2 cell monolayers from apical (AP) to basolateral (BL) side at 37 °C (*n* = 3). **p < .01, ^ns^ *p* > .05. (C) Transmission electron microscope images of basolateral medium collected after 3 h of incubation of Caco-2 cell monolayers with PTX/Mix-PMs. (D) Fluorescence emission spectra of basolateral medium collected after 3 h of incubation of Caco-2 cell monolayers with FRET micelles at excitation wavelength of 488 nm. (E) Transport of PTX across Caco-2 cell monolayers after 3 h of incubation with PTX/Mix-PMs under different conditions (*n* = 3). *P*_app_ of PTX (% of control) indicated the percentage of *P*_app_ to the control in the absence of any inhibitor at 37 °C. ^ns^ *p* > .05, *p < .05, and **p < .01 compared with the control.

In order to make sure whether the viability of Caco-2 cells is affected by PTX/PV-PMs and PTX/Mix-PMs, an evaluation of cytotoxicity of PTX/PV-PMs and PTX/Mix-PMs was further conducted. A relative cell viability ranging from (100.4 ± 4.4)% to (104.5 ± 7.0)% was observed for PTX/PV-PMs and from (101.2 ±  5.6)% to (105.5 ± 5.4)% for PTX/Mix-PMs below 50 μg/mL of PTX concentration (Figure 4(A)), suggesting that the cytotoxicity of PTX/PV-PMs and PTX/Mix-PMs against Caco-2 cells could be ignored at PTX concentration lower than 50 μg/mL.

To evaluate the effect of polymeric micelles on the enterocytes transport of PTX, the apparent permeability coefficient (*P*_app_) of PTX from AP to BL side of Caco-2 cell monolayers after treatment with Taxol^®^, PTX/PV-PMs and PTX/Mix-PMs were determined at concentration of 20 μg/mL PTX for all tested samples. As depicted in Figure 4(B), no significant difference in *P*_app_ was observed between PTX/PV-PMs ((0.71 ±  0.04) × 10^−6 ^cm/s) and Taxol^®^ ((0.60 ± 0.12) × 10^−6 ^cm/s) (*p* > .05), however, the *P*_app_ for PTX/Mix-PMs ((1.10 ± 0.08)  × 10^−6 ^cm/s) was highly significantly higher than that for Taxol^®^ and PTX/PV-PMs (*p* < .01), respectively. *P*_app_ value for PTX/PV-PMs and PTX/Mix-PMs was about 1.19- and 1.84-fold greater than that for Taxol^®^, respectively, indicating that polymeric micelles could evidently enhance PTX transport through Caco-2 cell mono layers, and Mix-PMs displayed better performance than PV-PMs to facilitate the transcellular transport of PTX.

Furthermore, no obvious change was detected in TEER values of Caco-2 cell monolayers during the period of the whole transport experiments (data not shown), suggesting that the integrity of Caco-2 cell monolayers was preserved and the transcellular transport of the micelles appeared not to proceed through paracellular pathway.

### Micelle integrity analysis

**Figure 5.**
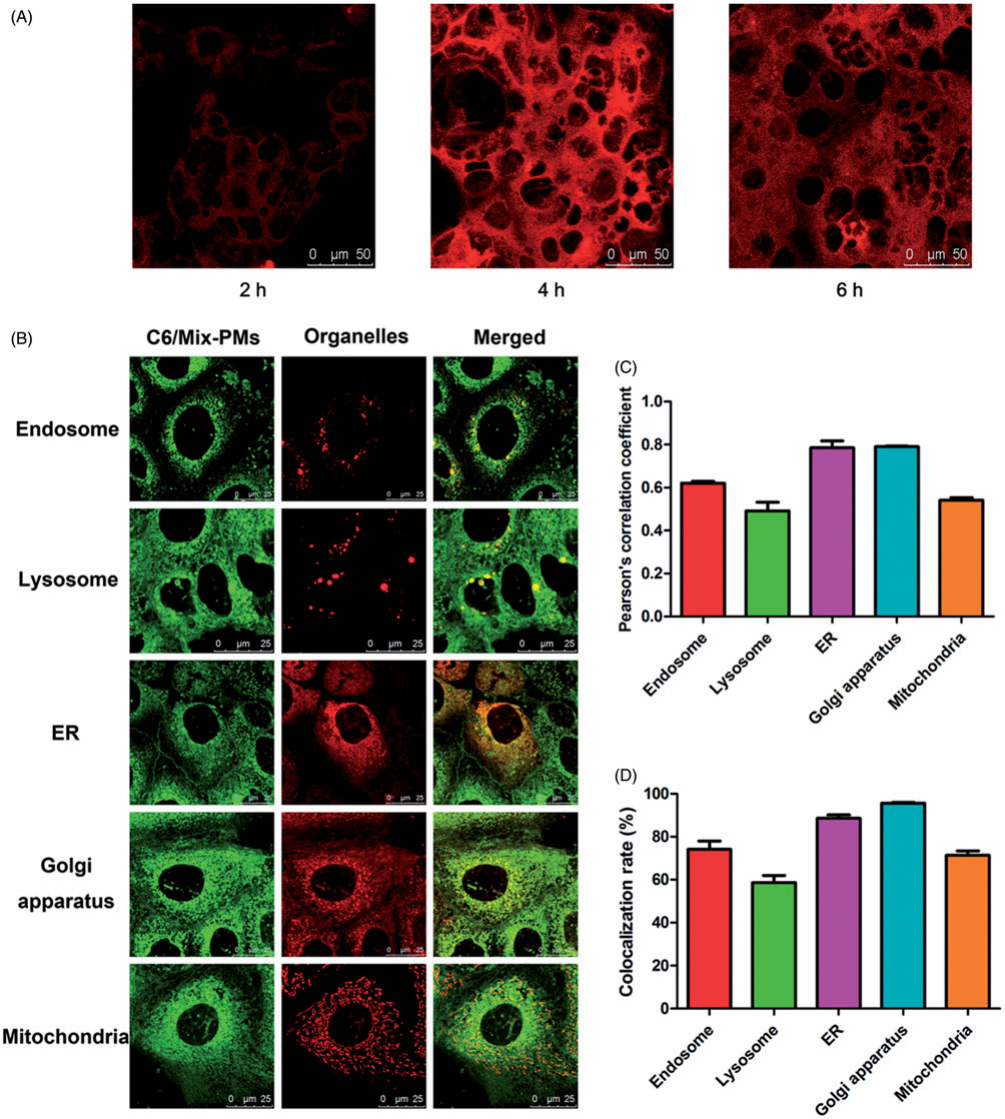
(A) Confocal images of Caco-2 cells in FRET lines after they were incubated with FRET micelles for different time at 37 °C. The Ex/Em of FRET line was DiO/DiI (488/568 nm). (B) CLSM images of colocalization of C6/Mix-PMs with different organelles after incubation of C6/Mix-PMs with Caco-2 cells for 1 h. Green, C6/Mix-PMs; red, specific organelle probes; yellow, colocalization of green and red signals. Late endosomes, lysosomes, endoplasmic reticulum (ER), Golgi apparatus and mitochondria were labeled with CellLight Late Endosomes-RFP, Lyso-Tracker Red, ER-Tracker Red, Golgi-Tracker BODIPY-TR ceramide, and Mito-Tracker Deep Red, respectively. Quantitative colocalization analysis of C6/Mix-PMs with different organelles by determining Pearson’s correlation coefficient (C) and colocalization rate (D).

Förster resonance energy transfer (FRET) method was used to evaluate the structure integrity of polymeric micelles after they were taken up by Caco-2 cells (Chen et al., 2008). The FRET pair DiO/DiI were therefore simultaneously loaded into the inner core of Mix-PMs (denoted as FRET micelles) with a diameter of 52.3 nm and PDI of 0.33 measured by DLS. As controls, DiO/Mix-PMs with 47.9 nm of diameter (PDI: 0.19) and DiI/Mix-PMs with 55.2 nm of diameter (PDI: 0.43) were also prepared. As known, if the micelles are intact, DiO and DiI are close enough (<10 nm) to each other for FRET to occur. In other words, at the excitation wavelength (488 nm) of the donor DiO, the acceptor DiI can emit fluorescence at 568 nm. On the other hand, the emission of DiI at 568 nm will extremely decrease upon release of DiO and/or DiI from the micelles. As shown in Figure 5(A), a strong FRET phenomenon was observed inside the cells, suggesting that the core-loaded molecules were encapsulated inside the micelles before they were internalized into cells, Mix-PMs were taken up by Caco-2 cells in the form of intact micelles and most of the micelles existed inside cells in intact micelles. Consequently, the behavior of the loaded probe in Caco-2 cells was considered to be the representative of probe-loaded micelles.

In addition, the fluorescence intensity in FRET lines increased with increasing incubation time in the first 4 h, and decreased thereafter, implying that cell uptake and accumulation of the micelles reached the maximum after 4 h of incubation. Consequently, the incubation time of 4 h was considered to be the optimal time for the subsequent studies on the intracellular trafficking of the micelles.

Besides, characterization of the basolateral media following transport experiments was extremely important to validate the transcytosis of the micelles from one side to the other of the monolayers. Thus, basolateral medium was collected after 3 h of incubation of Caco-2 cell monolayers with PTX/Mix-PMs, and examined by TEM. As shown in Figure 4(C), spherical nanoparticles, which were the same as those presented in Figure 2(D), could also be clearly observed in the basolateral side, proving that the intact micelles could transport across Caco-2 cell monolayers from one side to the other without disassembling inside cells.

Next, in order to make sure whether the core-loaded drug is absorbed through the intestinal membrane in its free form or in intact micelles, the fluorescence emission spectra of the basolateral medium collected after 3 h of incubation of Caco-2 cell monolayers with FRET micelles was detected at the excitation wavelength of 488 nm. As expected, a strong FRET phenomenon was clearly observed (Figure 4(D)). At the excitation wavelength of 488 nm for the donor DiO, the acceptor DiI could emit fluorescence at 568 nm, implying the energy transfer from donor DiO to acceptor DiI. In contrast, the emission of DiI at 568 nm was almost absent for the basolateral medium diluted in methanol. These indicated that the core-loaded molecules were still inside micelles after they were transported across Caco-2 cell monolayers. These findings suggested that most of the core-loaded drug was absorbed through the intestinal membrane in intact micelles.

### Transcytosis mechanism of mix-PMs

In order to investigate whether the transcellular transport of Mix-PMs was an active process, the *P*_app_ for PTX/Mix-PMs was determined at 4 °C by treating Caco-2 cell monolayers with PTX/Mix-PMs. As shown in Figure 4(E), the *P*_app_ value for PTX/Mix-PMs was significantly reduced to about 39.0% on lowering the incubation temperature from 37 to 4 °C (*p* < .01), strongly suggesting that the transcellular transport of PTX/Mix-PMs was an energy-dependent active process. On this basis, we posited that transcytosis was a prominent pathway for transcellular transport of PTX/Mix-PMs.

To further delineate the specific transport pathways involved in the transcellular transport of PTX/Mix-PMs, Caco-2 cell monolayers were pre-treated with biochemical inhibitors of various transport pathways. As shown in Figure 4(E), the *P*_app_ value for PTX/Mix-PMs was decreased to about 47.3% in the presence of chlorpromazine. Both M*β*CD and geinistein could evidently reduce the *P*_app_ to about 50.5 and 54.9%, respectively. However, macropinocytosis inhibitor amiloride had no significant effect on *P*_app_ value for PTX/Mix-PMs. Thus, it could be concluded that both clathrin- and caveolae/lipid rafts-mediated pathways were involved in transcellular transport of PTX/Mix-PMs and there was no apparent direct role for macropinocytosis-dependent mechanism.

### Intracellular trafficking progression of mix-PMs

To better understand the participation of the organelles in transcellular transport progression of Mix-PMs, involvement of different organelles was then investigated by examining the intracellular localization of C6/Mix-PMs with different organelles using CLSM following their internalization. The organelles, including late endosomes, lysosomes, endoplasmic reticulum (ER), Golgi apparatus, and mitochondria were stained using specific organelle trackers (red fluorescence). The colocalization of the internalized C6/Mix-PMs with these organelles was clearly observed (Figure 5(B)), respectively. In addition, the colocalization of C6/Mix-PMs with different organelles was further compared quantitatively with Pearson’s correlation coefficient (Figure 5(C)) and colocalization rate (Figure 5(D)), the index of colocalization extent between image pairs. As expected, the Pearson’s correlation coefficient was lower than 0.5 for lysosomes, indicating colocalization of the micelles with lysosomes was relatively poor. This suggested that a great portion of C6/Mix-PMs efficiently escaped from lysosomes before 1 h, or the intracellular trafficking of a very small portion of C6/Mix-PMs involved in lysosomes. The relatively low Pearson’s correlation coefficient for mitochondria (about 0.54) indicated that most of the micelles were not prone to transport to mitochondria. Different from lysosomes and mitochondria, the Pearson’s correlation coefficient for endosomes, ER and Golgi apparatus was remarkably higher than 0.5, implying that they were involved in the intracellular trafficking of C6/Mix-PMs. The changing trend of colocalization rate (Figure 5(D)) was approximately consistent with Pearson’s correlation coefficient (Figure 5(C)).

Moreover, it has been demonstrated that ER and Golgi apparatus are vital components for secretory ER/Golgi pathway and endocytic recycling pathway (Burd, 2011). Therefore, to better understand whether ER-to-Golgi apparatus pathway is existed in the intracellular trafficking progression of the micelles, we then investigated the effect of brefeldin A, a specific progression inhibitor for the secretory pathway from ER to Golgi apparatus by retrograding transport of enzymes of Golgi apparatus back to ER and inhibiting the anterograde movement of membrane beyond the mixed ER/Golgi apparatus system (Lippincott-Schwartz et al., 1989; Klausner et al., 1992; Tomas et al., 2010), on the transcellular transport of PTX/Mix-PMs. As shown in Figure 4(E), brefeldin A resulted in a remarkable inhibition of exocytosis of PTX/Mix-PMs (reduction of *P*_app_ to about 62.5%), suggesting the involvement of ER-to-Golgi apparatus pathway in their intracellular trafficking progression.

### Analysis of proteins involved in transcytosis of mix-PMs and finally excreted

The collected proteins for Mix-PMs-treated group and their homing information obtained from UniProtKB database were listed in Table 1. 12 proteins were identified in basolateral media after treatment of Caco-2 cell monolayers with Mix-PMs, in which several proteins localizing to typical organelles were found, suggesting the possible involvement of these organelles in intracellular trafficking of Mix-PMs.

**Table 1.** The collected proteins from basolateral media and their homing information for Mix-PMs-treated group.

Accession	Description	Score	Subcellular location
P06753-2	Isoform 2 of tropomyosin alpha-3 chain	73.52	Cytoplasm, Cytoskeleton
P09525	Annexin A4	72.06	Cytoplasm, exosome, Vesicle membrane, Plasma membrane
P07148	Fatty acid-binding protein	63.83	Cytoplasm, Apical cortex
H0YMD0	Annexin (Fragment)	53.01	/
H7C469	Uncharacterized protein (Fragment)	33.85	Lysosome
G3V1A4	Cofilin 1	26.03	Actin cytoskeleton
P30086	Phosphatidylethanolamine-binding protein 1	23.36	Cytoplasm, exosome
B7Z8M7	Ras-related protein Rab-1A	23.12	Intracellular
P40296	Malate dehydrogenase	22.58	Mitochondrion, Plasma membrane, exosome
P23141-3	Isoform 3 of liver carboxylesterase 1	22.2	Endoplasmic reticulum lumen
P02766	Transthyretin	20.41	Cytoplasm, secreted, exosome
O95633-2	Isoform 2 of follistatin-related protein 3	20.28	Golgi apparatus

## Discussion

We designed new mixed polymeric micelles by combination of PEOz-VES with TPGS1000 for enhancing intestinal absorption of PTX and elucidated their transcellular transport mechanisms and intracellular trafficking pathway. The developed mixed micelles were expected to take advantages of better diluting stability and pH-sensitivity of PEOz-VES micelles and absorption enhancement of TPGS1000 to improve transcellular transport of PTX and to accelerate PTX release inside the tumor cells.

First, PEOz-VES was successfully synthesized and utilized as the main micelle-forming component, and its favorable biocompatibility was further evidenced Figure 3(E). Previous studies showed that PEOz-PLA micelles exhibited remarkable pH-dependent drug release behavior resulted from ionization of PEOz chain located in outer shell of the micelles (Wang et al., 2005; Gao et al., 2015a; Zhao et al., 2015), which was further confirmed by the results of PV-PMs presented in Figure 3(A). This drug release behavior of the micelles was highly advantageous for targeted cancer therapy owing to significant suppression of the premature leakage of drug encapsulated in micelles during circulation in the bloodstream and rapid drug release at tumor sites and inside the tumor cells, thereby providing a sufficient amount of free drug to rapid and effectively kill cancer cells. However, if the micelles are used as oral delivery systems of antitumor drugs, especially BCS Class IV drugs and P-gp substrates, an effort should be made to ensure that the drugs are still encapsulated in micelles when they transport across the intestinal epithelium, which is the first barrier for oral delivery of antitumor drugs to tumor sites. We therefore set out to construct mixed micelles by insertion of TPGS1000 into PV-PMs on the basis of the premise of ensuring dilution stability, a certain degree of pH-sensitivity and absorption enhancement. As expected, the combination of PEOz-VES with TPGS1000 in a mass ratio of 1:1 exhibited low CMC (Supplementary Figure S1B) and negligible cytotoxicity up to 12 mg/mL polymers (Figure 3(E)), and drug-loaded Mix-PMs transportanted across the monolayers in the form of integrity Figures 4(C) and 5(A). What’s more, Mix-PMs presented not only much slower and sustained drug release but also retained pH-dependent drug release behavior (Figure 3(B)). Notably, both PTX/PV-PMs and PTX/Mix-PMs displayed much slower release rate in SIF with lower ionic strength than in PBS with higher ionic strength. A possible explanation for this behavior might be attributed to the difference in ionic strength in the two release media. As known, there exists a hydrated layer in PEOz-formed outer shell of the micelles. This hydrated layer may slow ionization progress of the tertiary amide groups along PEOz chain at pH lower than its p*K*_a_. Thinner hydrated layer is advantageous for tertiary amide groups to ionize. The strong electricity interaction between ions in the medium and water dipoles in hydrated layer might help to thin the hydrated layer, leading to acceleration of ionization of the tertiary amide group at pH lower than its p*K*_a_ and thereby accelerating drug release. Higher ionic strength, stronger the effect. Further, PTX/Mix-PMs showed better physicochemical characteristics including about 20 nm in diameter and positive charge (Supplementary Table S2), which are beneficial to their cell uptake (Jin et al., 2009) and higher drug loading. Thus, the designed mixed micelles might have high potential to oral delivery of antitumor drugs.

On the other hand, it was worth noting that the diameter of Mix-PMs was much smaller than that of PV-PMs (Supplementary Table S2), which might be assigned to the disparity in hydrophilicity between PEOz and PEG. As known, the balance between the attractive interaction of hydrophobic segments and the repulsive interaction of hydrophilic segments is required for micelle formation. For PEOz-VES polymers, PEOz moderates the association of PEOz-VES molecules, leading to micelle formation. Whereas for TPGS1000, PEG acts as PEOz. Compared with PEG, the ability of PEOz segment to moderate the association of the separating VES molecules might be poor due to the fact that the hydrophilicity of PEOz is stronger than that of PEG (Zalipsky et al., 1996; Wang & Hsiue, 2003), bigger agglomerates are therefore formed for PEOz-VES polymers. Hence, insertion of TPGS1000 promoted the formation of PEOz-VES micelles, leading to reduction of the micelle size.

It is well known that oral drugs need to transport through intestinal epithelium, which is the first barrier to their absorption, to be absorbed into systemic circulation. Therefore, transport of PTX/Mix-PMs through Caco-2 cell monolayer was investigated to evaluate the ability of Mix-PMs to permeate the epithelium layer. The PTX encapsulated in PV-PMs and Mix-PMs was transported across Caco-2 cell monolayers at a *P*_app_ value of 1.19- and 1.84-fold higher than that of Taxol^®^ (Figure 4(B)), respectively, and Mix-PMs displayed higher transport rate compared with PV-PMs. These findings were partially evidenced by the results of *in situ* intestinal absorption (Figure 3(D)). A possible explanation for this phenomenon might be assigned to enhancement of PTX solubility, involvement of multiple transcytosis pathway (Figure 4(E)) and alleviation of P-gp mediated efflux through masking effect by the micelles and inhibition of P-gp activity by their components (Li et al., 2014).

Understanding of transcytosis mechanisms may help us to rationally design nanoparticles and predict their location and distribution inside cells. Thus, we investigated the transport mechanism of Mix-PMs through Caco-2 cell monolayers. In general, pathways of nanoparticles transported through intestinal epithelium include paracellular transport and transcellular transport (He et al., 2013). In current study, the paracellular transport of Mix-PMs through opening tight junctions was excluded by unchangeable TEER values of Caco-2 cell monolayers during the whole period of transport experiments. Thus, we could assume that the micelles could cross the intestinal barrier predominantly by a transcellular pathway. Further, remarkable reduction of PTX/Mix-PMs transport by lowering temperature from 37 °C to 4 °C was an indication for the involvement of an active and energy-dependent process for the transcellular transport of Mix-PMs (Figure 4(E)). Additionally, Mix-PMs were validated to cross the monolayers in the form of integrity by both TEM (Figure 4(C)) and FRET intuitive observations (Figures 4(D) and 5(A)).

**Scheme 1.**
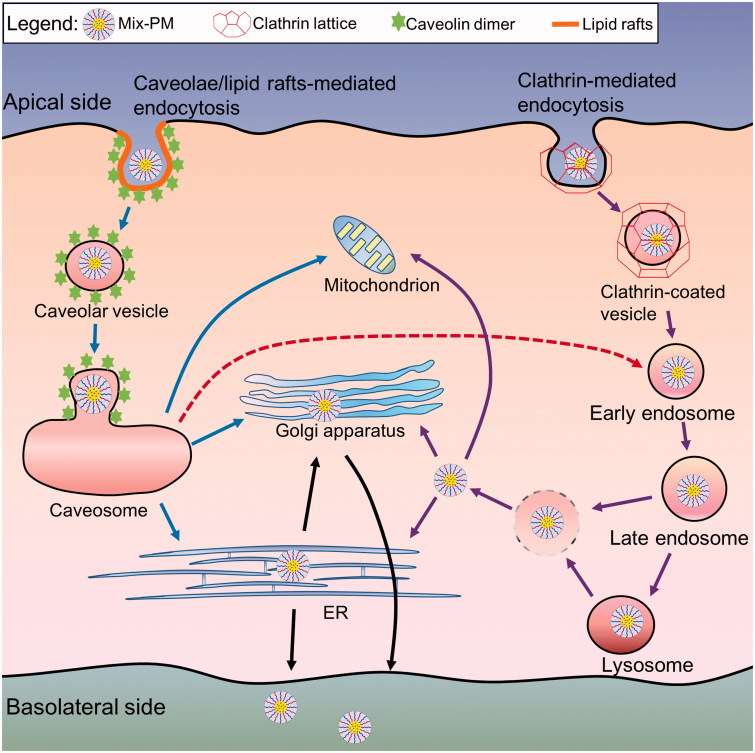
Schematic diagrams depicting predicted transcellular transport pathways and intracellular trafficking routes of Mix-PMs through Caco-2 cell monolayers.

As reported, clathrin- and caveolae-mediated process, clathrin- and caveolae-independent pathway, and micropinocytosis have been characterized as distinct transcytosis pathways of nanoparticles and macromolecules (Conner & Schmid, 2003; Singh et al., 2010). The transport pathway of nanoparticles is influenced by many factors such as particle size, shape, surface chemistry and composition (Gratton et al., 2008). Our mechanistic studies provided important convincing evidence for transmembrane transport mechanism of Mix-PMs based on the effects of various specific inhibitors of selected transcytosis pathways on transcellular transport of Mix-PMs. Pre-treatment of the monolayers with amiloride had no effect on *P*_app_ of the micelles, however, *P*_app_ in the presence of chlorpromazine, M*β*CD and genistein was effectively decreased (Figure 4(E)). We therefore inferred that the transcytosis of Mix-PMs was regulated by combination of multiple transport mechanisms including clathrin- and caveolae/lipid rafts-mediated pathways (Scheme 1). None of the specific inhibitors led to complete inhibition (no more than 80% in Figure 4(E)) of transcellular transport, most likely indicating the role of clathrin- and caveolae-independent pathways for transcytosis, which should be further identified.

In general, nanoparticles employ endocytic vesicles and utilize complex trafficking machinery to localize to various organelles (Yu et al., 2013). For transcellular transport, the nanoparticles are first engulfed in membrane invaginations forming endosomes or caveosomes, then delivered by endosomes or caveosomes to various organelles including lysosomes, ER and Golgi apparatus, and finally cross polarized cells such as Caco-2 cells (transcytosis) (Singh et al., 2010). As known, the intracellular trafficking of nanoparticles is dependent on their endocytosis pathway besides surface properties and composition (Gratton et al., 2008). The present study provided a more complete understanding of intracellular trafficking routes of Mix-PMs by monitoring the intracellular colocalization of C6-labeled Mix-PMs with various organelles by CLSM. CLSM images revealed that late endosomes, lysosomes, ER and Golgi apparatus were all involved in intracellular trafficking of Mix-PMs (Figure 5(B)). This was consistent with the results of transcytosis mechanisms (Figure 4(E)). On the other hand, ER-to-Golgi transfer was involved in intracellular transport pathway of Mix-PMs (Figure 4(E)). Moreover, different from classical organelles like endosomes, lysosomes, Golgi apparatus and ER, mitochondria mainly function as energy-supplier but do not involve in the intracellular trafficking of macromolecules and aggregates in general (He et al., 2013). However, it was observed that a small number of micelles were distributed to mitochondria (Figure 5(B)), which might be assigned to the specific interaction of TPGS1000 with mitochondria (Yu et al., 2015). Thus, it could be inferred that Mix-PMs, transported across Caco-2 cell monolayers by clathrin-mediated transcytosis, were first internalized by Caco-2 cells through endocytosis, transported to late endosomes, followed by escape for the most from late endosomes and fusion for the other with lysosomes followed by escape from lysosomes. Then the escaped Mix-PMs were distributed to ER, Golgi apparatus and mitochondria followed by transfer of some micelles distributed to ER to Golgi apparatus (Scheme 1). While the other intracellular trafficking route also exist simultaneously: Mix-PMs, transported across Caco-2 cell monolayers through caveolae/lipid rafts-mediated transcytosis, transported from caveosomes directly to ER, Golgi apparatus and mitochondria followed by transfer from ER to Golgi apparatus (Scheme 1). Another possible intracellular trafficking route of Mix-PMs following endocytosis mediated by caveolae/lipid rafts could not be overlooked: Mix-PMs transported from caveosomes to early endosomes and the subsequent progression was the same to the first possible route mentioned above (Scheme 1) (Mosesson et al., 2008; Kiss & Botos, 2009). Finally, Mix-PMs were excreted from Caco-2 cells by exocytosis secretion to cross Caco-2 cell monolayers.

As known, some proteins are responsible for intracellular transport of lipids, block copolymers and nanoparticles (Sakai-Kato et al., 2014). To elucidate the proteins involved in transport of objects in cells, the common method is to use siRNAs to down-regulate the expression of these specific transport proteins. However, we need to know the possible involved transport proteins at first. To date, little is known about proteins involved in transmembrane transport of nanoparticles. In order to screen the possible proteins involved in transport of transcellular transfer of the micelles, we hypothesized that some of these specific proteins might excrete from the cells following treatment of Caco-2 cell monolayers with micelles. Favorably, 12 proteins were collected from basolateral media, separated and analyzed, suggesting that these proteins might be related to transcellular transport of the micelles. Further studies are needed to verify whether these proteins are responsible for transcellular transport of the micelles by using siRNAs to down-regulate their expression. Notably, there are some other transport proteins not excreted from the cells to basolateral media. Furthermore, it was worth noting that there were correlations of the colocalization results of CLSM observation (Figure 5(B)) with the subcellular location of the collected proteins (Table 1). This finding is specific to these micelles. It will be necessary to elucidate the intracellular trafficking and proteins involved in transcytosis for different nanoparticles.

## Conclusions

The newly mixed micelles Mix-PMs were prepared by combination of the synthesized PEOz-VES with TPGS1000 and characterized for intestinal delivery of water insoluble drug PTX. We demonstrated that the developed Mix-PMs exhibited excellent performance featured by smaller size, higher encapsulation efficiency, desired pH-sensitivity, favorable cytocompatibility, and enhanced transcellular transport efficiency compared with PV-PMs. Mechanistic studies demonstrated that Mix-PMs were transported across Caco-2 cell monolayers in the form of integrity mainly through both clathrin- and caveolae/lipid rafts-mediated transcytosis. In addition, the intracellular trafficking progression of Mix-PMs was identified, and the proteins involved in transcytosis of Mix-PMs and finally excreted were roughly disclosed. These results suggested that Mix-PMs developed in the present study could be a promising nanocarrier for intestinal delivery of water insoluble drugs. The present study also evidenced that the newly synthesized pH-sensitive polymer PEOz-VES exhibited favorable biocompatibility and could self-assemble into nanosized micelles, which make it possible to be a potential encapsulant in anticancer drug delivery domains.

## Supplementary Material

IDRD_Liu_et_al_Supplemental_Content.docx
