# Supplementary material for: Improved intestinal absorption of paclitaxel by mixed micelles self-assembled from vitamin E succinate-based amphiphilic polymers and their transcellular transport mechanism and intracellular trafficking routes
Source: Drug Deliv. 2018 Jan 9;25(1):210–25. doi: 10.1080/10717544.2017.1419513 (PMC6058530; doi:10.1080/10717544.2017.1419513)
Supplement: IDRD_Liu_et_al_Supplemental_Content.docx [file IDRD_A_1419513_SM4933.docx]

**Supplemental Material**


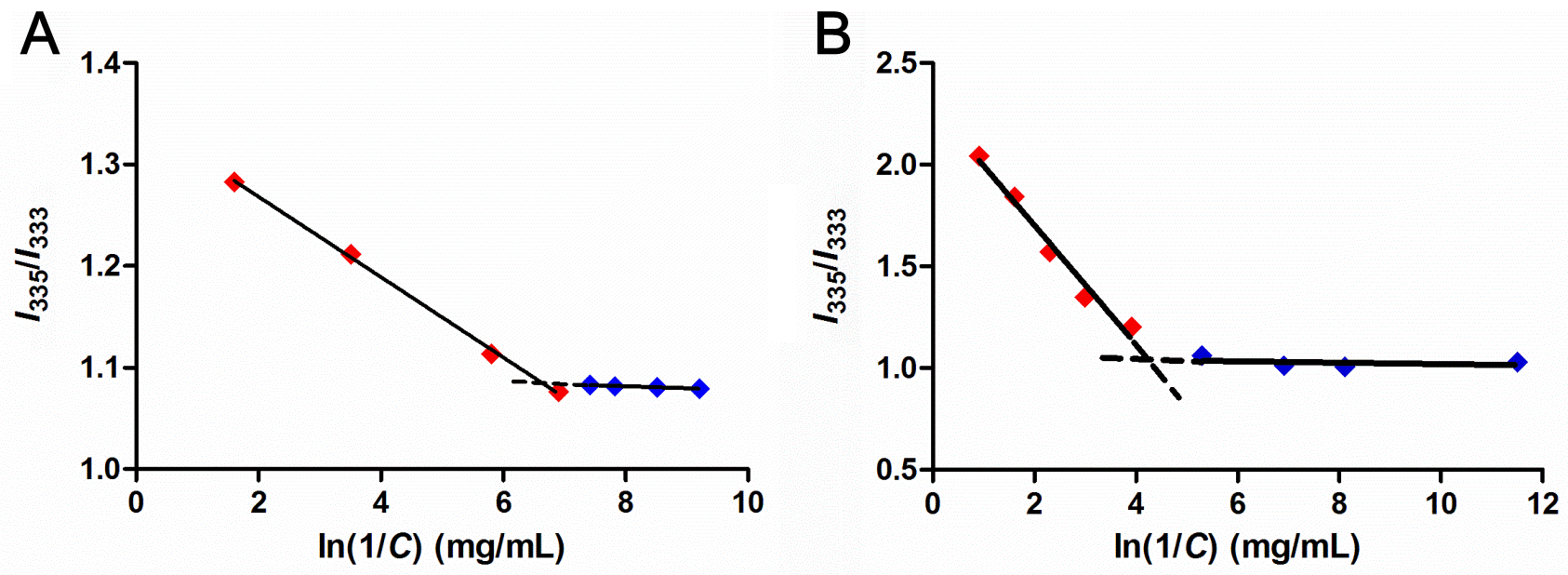


**Figure S1.** Plots of *I*_335_/*I*_333_ from excitation of pyrene as a function of ln(1/*C*) for PEOz-VES (A) and a mixture of PEOz-VES with TPGS1000 in a mass ratio of 1:1 (B) in distilled water.


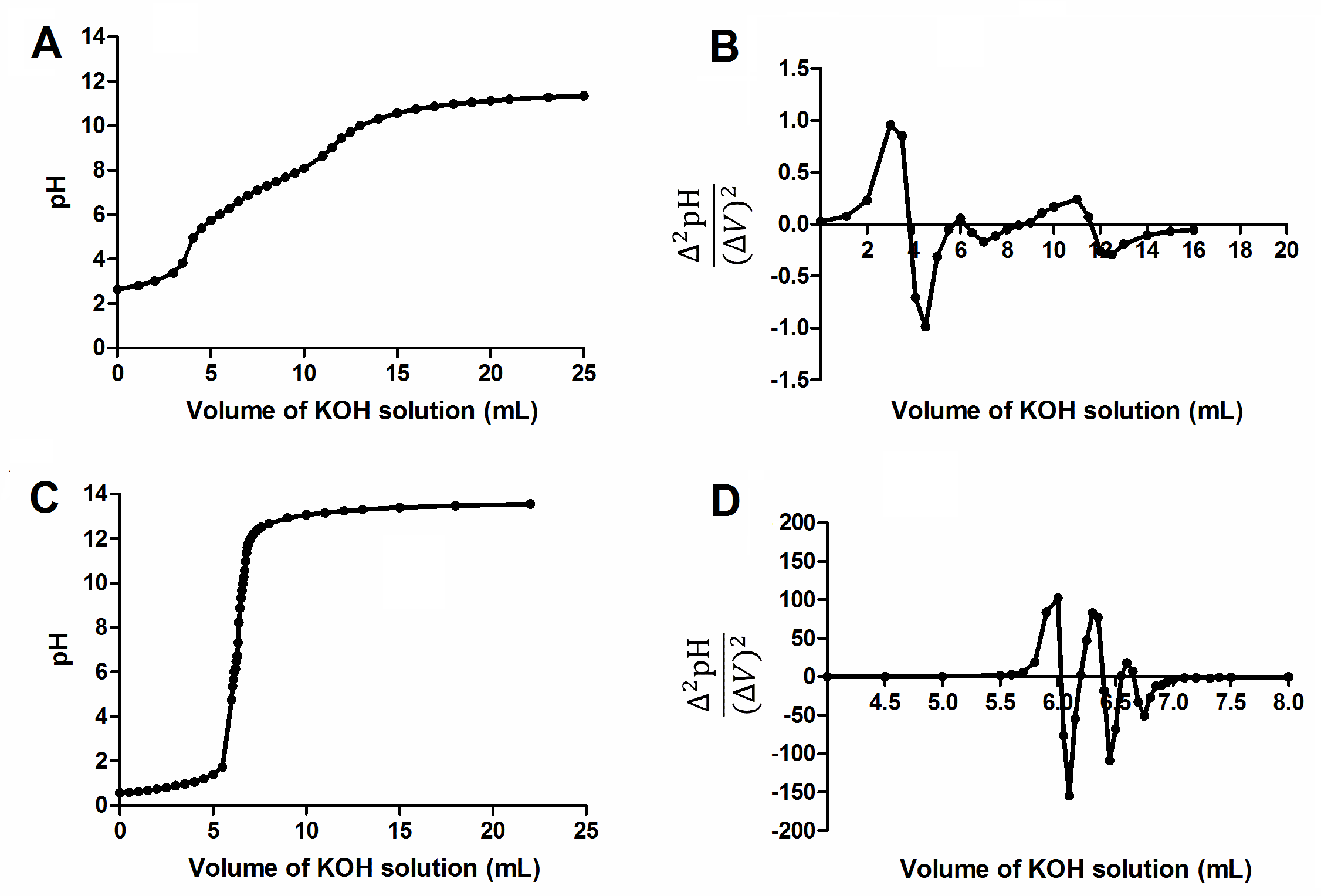


**Figure S2.** Acid-base titration profile (A) and its second-order differential curve (B) of PEOz-NH_2_. Acid-base titration profile (C) and its second-order differential curve (D) of PEOz-VES.

**Table S1.** The organelle trackers and their concentration used in the experiment.

| **Organelle trackers** | **Added amount or final concentration** | **Organelle** |
| --- | --- | --- |
| CellLight Late Endosome-RFP | 5 μL | Endosomes |
| Lyso-Tracker Red DND-99 | 150 nM | Lysosomes |
| ER-Tracker Red | 1 μM | Endoplasmic reticulum |
| Golgi-Tracker BODIPY-TR ceramide | 5 μM | Golgi apparatus |
| Mito-Tracker Deep Red | 400 nM | Mitochondria |

**Table S2.** Physicochemical characteristics of PTX-loaded polymeric micelles (*n*=3).

| **Micelles** | **Diameter**  **(nm)** | **PDI** | **Zeta potential**  **(mV)** | **LC**  **(%)** | **EE**  **(%)** |
| --- | --- | --- | --- | --- | --- |
| PTX/PV-PMs | 30.2±1.4 | 0.51±0.01 | 4.86 | 2.63±0.16 | 84.14±3.38 |
| PTX/Mix-PMs | 20.4±0.3*** | 0.30±0.01 | 1.42 | 3.25±0.18** | 95.81±4.16** |

***p*<0.01, ****p*<0.001 vs. PTX/PV-PMs.
